# COVID-19 Vaccination Coverage, Behaviors, and Intentions among Adults with Previous Diagnosis, United States 

**DOI:** 10.3201/eid2803.211561

**Published:** 2022-03

**Authors:** Kimberly H. Nguyen, Jing Huang, Kathrine Mansfield, Laura Corlin, Jennifer D. Allen

**Affiliations:** Tufts University School of Medicine, Boston, Massachusetts, USA (K.H. Nguyen, J. Huang, K. Mansfield, L. Corlin);; Tufts University School of Engineering, Medford, Massachusetts, Massachusetts, USA (L. Corlin);; Tufts University, Medford (J.D. Allen)

**Keywords:** COVID-19, 2019 novel coronavirus disease, coronavirus disease, severe acute respiratory syndrome coronavirus 2, SARS-CoV-2, viruses, respiratory infections, zoonoses, COVID-19 vaccine, vaccine hesitancy, vaccine confidence, United States

## Abstract

To determine the extent of gaps in coronavirus disease (COVID-19) vaccine coverage among those in the United States with and without previous COVID-19 diagnoses, we used July 21–August 2, 2021, data from a large, nationally representative survey (Household Pulse Survey). We analyzed vaccine receipt (≥1 dose and full vaccination) and intention to be vaccinated for 63,266 persons. Vaccination receipt was lower among those who had a prior diagnosis of COVID-19 compared to those without: >1 dose: 73% and 85%, respectively, p<0.001; full vaccination: 69% and 82%, respectively, p<0.001). Reluctance to be vaccinated was higher among those with a previous COVID-19 diagnosis (14%) than among those without (9%). These findings suggest the need to focus educational and confidence-building interventions on adults when they receive a COVID-19 diagnosis, during clinic visits, or at the time of discharge if hospitalized and to better educate the public about the value of being vaccinated, regardless of previous COVID-19 status.

The goal of the US coronavirus disease (COVID-19) vaccination campaign is to substantially reduce the overall burden of COVID-19 by preventing severe acute respiratory syndrome coronavirus (SARS-CoV-2) infections, reducing virus transmission, and reducing hospitalizations and deaths. Data from the Centers for Disease Control and Prevention (CDC) have demonstrated that the number of COVID-19 patients in intensive care units is higher in states with the lowest vaccination levels than in states with highest vaccination levels (*1*,*2*). However, as of September 10, 2021, ≈15% of US adults were not vaccinated, and 28% were not fully vaccinated (*3*).

Whereas reasons for nonvaccination or undervaccination are multifactorial (*4*–*8*), studies suggest that persons with a previous diagnosis of COVID-19 are less likely to be vaccinated than are those who have not previously had COVID-19 (*9*). However, CDC recommends that persons previously infected with SARS-CoV-2 still get the vaccine (*10*). This recommendation reflects the knowledge that although the rate of reinfection among persons with previous COVID-19 illness is very low (*11*–*13*), natural immunity from infection may not provide a sufficient level of protection, particularly among the elderly (*14*). Persons who have had COVID-19 can still become severely ill if reinfected, and even those who were initially asymptomatic can have ongoing health problems several weeks or even longer after getting reinfected (long haulers) (*10*). Moreover, those who were previously infected with SARS-CoV-2 and became infected again can still transmit the infection to others (*10*). Vaccination not only protects persons who have not been previously infected but also provides a strong boost in protection for those who have recovered from COVID-19 (*10*); a growing body of evidence demonstrates added protection against reinfection for persons who were previously infected with SARS-CoV-2 when they have a higher titer of antibodies resulting from vaccination (*15*). It is vital that all persons be fully vaccinated, regardless of infection history. Without achieving this level of vaccination coverage, COVID-19 spikes and clusters will probably re-emerge in areas with low vaccination levels.

Vaccination coverage and intentions to be vaccinated among persons who had a previous diagnosis of COVID-19 is unknown. Our goals with this study were to 1) compare vaccination coverage (>1 dose and receipt of all recommended doses) and intention to be vaccinated, by previous COVID-19 status; 2) examine factors associated with vaccination coverage and intention to be vaccinated and reasons for nonvaccination, by previous COVID-19 status; and 3) assess the correlation between state-level prevalence of previous COVID-19 diagnoses and COVID-19 vaccination coverage, by using data from a large, nationally representative household survey. Knowing the extent of gaps in vaccination coverage among those with and without a history of COVID-19, as well as reasons for these gaps, is necessary for designing and targeting effective interventions to improve vaccine uptake at the population level.

## Methods

### Survey Design

To help elucidate household experiences during the COVID-19 pandemic, we examined data from the Household Pulse Survey (HPS), a large, nationally representative household survey that has been conducted by the US Census Bureau since April 2020 (*16*). The study design of the HPS has been published (*17*). We examined data collected during July 21–August 2, 2021; a total of 63,266 persons responded (response rate 6.1%) (*18*). This study was reviewed by Tufts University Health Sciences Institutional Review Board and was not considered human subjects research.

### COVID-19 Questions

HPS questions cover COVID-19 diagnosis, vaccination coverage, vaccination intention, and reasons for not being vaccinated. COVID-19 diagnosis was assessed by the following question: “Has a doctor or other health care provider ever told you that you have COVID-19?” (yes/no/not sure). Because of the low numbers of responses in the not sure category (<1%), this study examined only responses for yes and no. COVID-19 vaccination receipt (>1 dose) was assessed with the following question: “Have you received a COVID-19 vaccine?” (yes/no). Adults who reported having received >1 dose were asked: “Did you receive (or do you plan to receive) all required doses?” (Yes, received all required doses/Yes, plan to receive all required doses/No, don’t plan to receive all required doses). Full vaccination coverage was defined as a response that all required doses have been received.

Among adults who did not receive any COVID-19 vaccinations, we assessed future vaccination intentions by asking, “Once a vaccine to prevent COVID-19 is available to you, would you... definitely, probably, be unsure about, probably not, or definitely not get(ting) a vaccine.” Because the vaccination intention questions were asked only of those who were not vaccinated, assessing intention over time would show bias as more persons got vaccinated (reducing the sample size of those who are asked about intention). To reduce this potential for bias, the denominator for vaccination intention was everyone in the sample, including those who were vaccinated. We categorized unvaccinated respondents who did not definitely plan to be vaccinated as uncertain (those who probably will get vaccinated or are unsure about getting vaccinated) or reluctant (those who probably will not or definitely will not get vaccinated). Because of the low numbers of respondents who definitely would get vaccinated (<5%) and their similarities to the vaccinated group, we did not include them in this study.

Unvaccinated persons who did not report that they would definitely get vaccinated were asked about their reasons for not getting vaccinated: “Which of the following, if any, are reasons that you [probably will/are unsure about/probably won't/definitely won't] get a COVID-19 vaccine/did not receive all required doses.” Response options, for which they could select all that applied: 1) “I am concerned about possible side effects of a COVID-19 vaccine,” 2) “I don't know if a COVID-19 vaccine will protect me,” 3) “I don't believe I need a COVID-19 vaccine,” 4) “My doctor has not recommended it,” 5) “I plan to wait and see if it is safe and may get it later,” 6) “I am concerned about the cost of a COVID-19 vaccine,” 7) “I don't trust COVID-19 vaccines,” 8)” I don't trust the government,” 9) “I don’t think COVID-19 is that big of a threat,” 10) “It’s hard for me to get a COVID-19 vaccine,” and 11) “Other (specify).” Respondents who reported that they were not fully vaccinated, despite already having received 1 dose, were given additional options: 1) “I believe one dose is enough to protect me,” and 2) “I experienced side effects from the dose of COVID-19 vaccine received.”

### Sociodemographic Characteristics

We assessed the following sociodemographic characteristics: age group (18–49 years/50–64 years/>65 years, gender (male/female/transgender or other), race/ethnicity (non-Hispanic White/non-Hispanic Black/Hispanic/non-Hispanic Asian/non-Hispanic other or multiple races), educational attainment (less than high school/some college or college graduate/above college graduate), annual household income (<$35,000/$35,000–$49,999/$50,000–$74,999/>$75,000/not reported), health insurance coverage (yes/no), number of persons in the household (1–2/3–5/>6), and housing structure (single-family home/condominium or townhouse/multi-unit housing/other).

### Analyses

We analyzed prevalence of previous COVID-19 infection overall and by sociodemographic characteristics. We determined the association between previous COVID-19 diagnosis and COVID-19 vaccination coverage (>1 dose and receipt of all required doses) by using multivariable regression analyses adjusted for sociodemographic variables (age group, gender, race/ethnicity, educational status, annual household income, insurance status, household size, and housing structure). We also examined factors associated with COVID-19 vaccination coverage (>1 dose and receipt of all required doses) stratified by previous COVID-19 disease status. Furthermore, intention to get vaccinated (uncertain/reluctant) was analyzed by previous COVID-19 disease status overall and by sociodemographic characteristics. We assessed factors associated with vaccination intention (uncertain/reluctant) stratified by previous COVID-19 disease status in multivariable analyses by using adjusted prevalence ratio (aPR). Reasons for not getting vaccinated were assessed by previous COVID-19 disease status. Proportions and 95% CIs for reasons for not getting vaccinated were examined by intention categories (uncertain/reluctant). We created a scatterplot of state-level prevalence of previous COVID-19 infection and vaccination coverage and determined R^2^ for the correlation between the 2 variables. We conducted contrast tests for the differences in proportions, comparing each category to the referent category and comparing those who ever and never had a COVID-19 diagnosis with a 0.05 significance level (α = 0.05). We used Stata 16.1 (*17*) to account for the survey design and weights to ensure a nationally representative sample. Unless otherwise noted, all results presented in this report are significant at p<0.05.

## Results

### Sample Characteristics

More than one half of the sample participants were 18–49 years of age, one quarter were 50–64 years, and 22% were >65 years (Table 1). Most (62%) were non-Hispanic White, 17% were Hispanic, 11% were non-Hispanic Black, 6% were non-Hispanic Asian, and 4% were non-Hispanic other/multiple race. More than 60% had at least some college education, 32% had annual household incomes of >$75,000, and most (92%) had health insurance. Half (50%) of the households had 3–5 persons living in the household, 39% had 1–2 persons, and 11% had >6 persons. Furthermore, 69% lived in single-family homes, 19% in a multi-unit home, 8% in a townhouse/condominium, and 5% in other settings (e.g., mobile homes, boats, vans, recreational vehicles).

**Table 1:** Prevalence of previous COVID-19 diagnosis among adults, by socioeconomic characteristics, United States, July 21–August 2, 2021*

Characteristic	Total, % (95% CI), N = 63,266	Ever had COVID-19 % (95% CI), n = 7,716	Never had COVID-19 % (95% CI), n = 55,186
All adults, >18 y		14.6 (14.1,15.2)	84.3 (83.7–84.9)
Age group, y			
18–49 (referent)	52.0 (51.7–52.3)	16.5 (15.4–17.6)	82.3 (81.2–83.4)
50–64	25.9 (25.5–26.2)	15.2 (14.2–16.2)	84.3 (83.3–85.2)
>65	22.2 (21.9–22.4)	9.5 (8.5–10.6)†	89.1 (87.9–90.1)
Sex			
F (referent)	50.6 (50.3–50.8)	15.2 (14.5–15.9)	84.2 (83.5–84.9)
M	46.9 (46.6–47.3)	13.9 (13.0–14.9)	85.1 (84.0–86.0)
Transgender or other	2.5 (2.2–2.8)	15.3 (11.5–20.2)	73.9 (67.9–79.1)
Race/ethnicity			
Non-Hispanic White (referent)	62.4 (62,2–62.6)	13.1 (12.6–13.7)	86.4 (85.8–86.9)
Non-Hispanic Black	11.0 (10.8–11.2)	15.9 (14.2–17.8)†	82.8 (80.6–84.7)
Hispanic	17.2 (17.0–17.4)	20.8 (18.9–22.9)†	76.3 (74.1–78.4)
Non-Hispanic Asian	5.6 (5.4–5.9)	8.8 (7.2–10.6)†	90.5 (88.6–92.1)
Non-Hispanic other/multiple races	3.8 (3.5–4.0)	15.4 (12.7–18.5)	82.8 (79.6–85.6)
Education			
High school or less (referent)	38.5 (38.4–38.7)	15.9 (14.8–17.2)	82.3 (81.0–83.6)
Some college or college graduate	47.5 (47.2–47.8)	14.8 (14.1–15.4)	84.6 (83.9–85.3)
Above college graduate	14.0 (13.7–14.2)	10.3 (9.6–11.1)†	88.8 (88.0–89.5)
Annual household income			
<$35,000 (reference)	20.0 (19.3–20.7)	13.7 (12.4–15.1)	84.9 (83.4–86.2)
$35,000–$49,999	8.9 (8.5–9.3)	15.2 (13.6–16.9)	84.1 (82.4–85.7)
$50,000–$74,999	12.7 (12.2–13.2)	16.0 (14.3,18.0)†	83.1 (81.0–85.0)
>$75,000	32.2 (31.6–32.7)	12.7 (11.9–13.4)	86.9 (86.1–87.6)
Did not report	26.3 (25.6–27.0)	16.8 (15.5–18.2)†	81.5 (79.9–82.9)
Insurance status			
Insured (reference)	91.8 (91.2–92.4)	13.6 (13.0–14.3)	85.6 (85.0–86.2)
Not insured	8.2 (7.6–8.8)	17.1 (14.0–20.6)†	79.9 (76.4–83.1)
No. persons in household			
1–2 (referent)	39.5 (38.6–40.4)	12.4 (11.8–13.0)	87.2 (86.6–87.8)
3–5	50.0 (49.2–50.7)	15.3 (14.4–16.1)†	83.9 (82.9–84.8)
>6	10.6 (9.8–11.4)	19.8 (17.3–22.5)†	75.8 (72.9–78.5)
Housing structure			
Single-family home (referent)	68.8 (68.1–69.6)	14.0 (13.4–14.7)	85.3 (84.6–86.1)
Townhouse/condo	7.6 (7.1–8.1)	12.1 (9.6–15.1)	86.3 (83.1–89.0)
Multi-unit home	18.6 (18.0–19.3)	13.7 (12.1–15.5)	85.1 (83.2–86.8)
Other: e.g., mobile home, boat, van, RV	5.0 (4.6–5.4)	15.0 (12.8–17.4)	81.9 (79.5–84.1)

*All percentages are weighted. COVID-19, coronavirus disease; RV, recreational vehicle. †Significant at p<0.05 comparing each group to the referent group for likelihood of having previously had COVID-19.

### COVID-19 Infection and Vaccine Receipt

Nationally, 15% of adults had a previous diagnosis of COVID-19 (Table 1). Prevalence of having a positive history of COVID-19 infection was highest among adults 18–49 years of age (17%), Hispanic adults (21%), and adults with high school education or less (16%) compared with their respective counterparts (Table 1). Moreover, respondents living in larger households were more likely to report having been infected with COVID-19 (20% among households with >6 persons) compared with those living in smaller households (12% among households with <2 persons).

Vaccination coverage (>1 dose and full vaccination) was lower among those who ever had COVID-19 than those who had no history of COVID-19 infection (Table 2). For example, those with a history of COVID-19 were 0.88 (95% CI 0.86–0.91) times as likely to get >1 COVID-19 vaccination and 0.86 (95% CI 0.84–0.89) times as likely to be fully vaccinated. Across all sociodemographic characteristics, vaccination coverage (>1 dose and full vaccination) was lower among those with a history of COVID-19 infection than among those who never had COVID-19 (Appendix Table 1). Among those with a history of COVID-19, factors associated with lower vaccination coverage (>1 dose) were being male (aPR 0.93, 95% CI 0.88–0.99) and living in larger households (>6 persons: aPR 0.87, 95% CI 0.77–0.99) compared with their respective counterparts. Being Hispanic (aPR 1.11, 95% CI 1.01–1.22), non-Hispanic Asian (aPR 1.21, 95% CI 1.10–1.32), having a high education level (above college degree: aPR 1.18, 95% CI 1.11–1.26), and high income (>$75,000: aPR 1.14, 95% CI 1.05–1.25) were associated with higher vaccination coverage (>1 dose) compared with their respective counterparts.

**Table 2:** Association between previous COVID-19 diagnosis and vaccination coverage, United States, July 21–August 2, 2021*

Prior COVID-19 diagnosis†	Received >1 dose			Received all required doses	
	% (95% CI)	aPR (95% CI)		% (95% CI)	aPR (95% CI)
Yes	73.3 (71.4–75.2)	0.88 (0.86–0.91)		68.9 (67.0–70.7)	0.86 (0.84–0.89)
No	84.6 (83.9–85.2)	Referent		81.6 (80.9–82.4)	Referent

*All percentages are weighted. aPR, adjusted prevalence ratio; COVID-19, coronavirus disease. †Multivariable regression model adjusting for age group, gender, race/ethnicity, educational attainment, annual household income, insurance status, household size, and housing structure.

Across all states, prevalence of previous COVID-19 infection was inversely proportional to COVID-19 vaccination coverage (R^2^ = 0.4074) (Figure). For example, in Mississippi, vaccination coverage was 75% and prevalence of previous COVID-19 infection was 22%, whereas in Vermont, vaccination coverage was 90% and prevalence of previous COVID-19 infection was 5%, and in Oregon, vaccination coverage was 88% and prevalence of previous COVID-19 infection was 7%.

**Figure:**
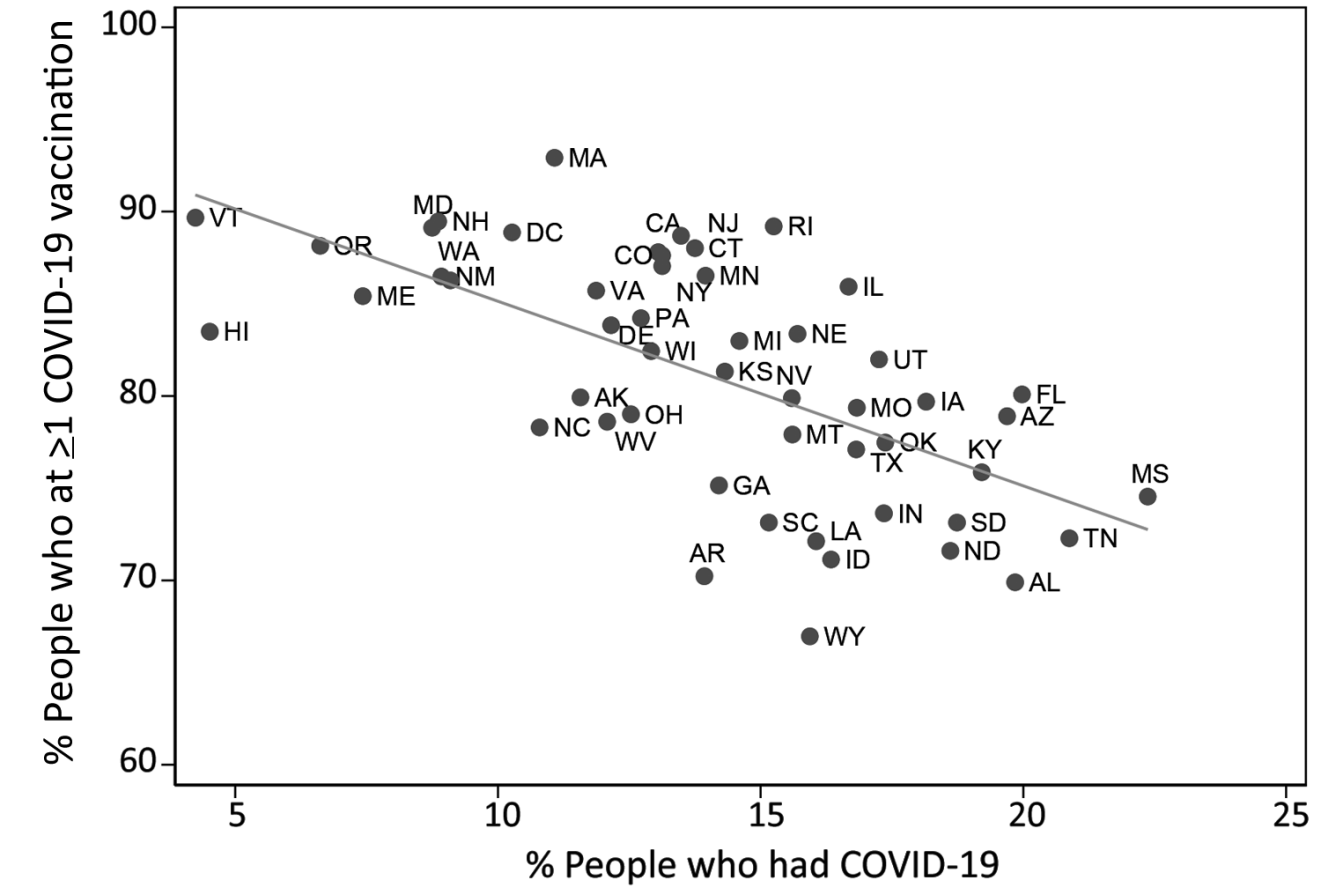
COVID-19 vaccination coverage estimates and prevalence of previous COVID-19 infection by state, United States, July 21–August 2, 2021. COVID-19, coronavirus disease.

### Vaccination Intentions

Intention to get vaccinated and factors associated with vaccination also differed by previous COVID-19 status (Appendix Table 2). The proportion of adults who were uncertain about vaccination was higher among those with a previous COVID-19 diagnosis (10%) than among those without (5%), and the proportion of adults who were reluctant about vaccination was higher among those with a previous COVID-19 diagnosis (14%) than among those without (9%). Across most socioeconomic characteristics, the proportion of uncertain and reluctant adults was also higher among those who ever had COVID-19 than among those who never had COVID-19. Furthermore, factors associated with being uncertain differed by COVID-19 case status. For example, being non-Hispanic Black was associated with being uncertain about getting vaccinated among those who never had COVID-19 (aPR 1.68, 95% CI 1.34–2.12) but not among those who ever had COVID-19. Furthermore, having high educational levels (above college graduate: aPR 0.29, 95% CI 0.22–0.38) and high income levels (>$75,000: aPR 0.61, 95% CI 0.50–0.74) were associated with lower risk of being reluctant to get vaccinated, and living in larger households (>6 persons: aPR 1.78, 95% CI 1.41–2.24) was associated with vaccination reluctance among those who never had COVID-19 but not among those who ever had COVID-19. Insurance status was associated with lower risk of being reluctant but did not differ by COVID-19 case history.

Reasons for not getting vaccinated differed among those with and without a previous COVID-19 diagnosis (Table 3). Among uncertain and reluctant adults, a higher percentage of respondents who ever had COVID-19, compared with those who never had COVID-19, reported concerns about possible side effects (71% vs. 57%), lack of doctor recommendation (15% vs. 9%), and other reasons (30% vs. 22%).

**Table 3:** Reasons for not getting vaccinated, by vaccination intention and stratified by previous COVID-19 diagnosis, United States, July 21–August 2, 2021*

Reason	Ever had COVID-19, % (95% CI)	Never had COVID-19, % (95% CI), referent
Probably/unsure		
Concerned about possible side effects	70.6 (62.8–77.4)†	57.2 (52.1–62.2)
Don’t know if a vaccine will protect me	20.7 (14.3–29.0)	20.5 (17.2–24.2)
Don’t believe I need a vaccine	5.4 (3.0–9.5)	8.8 (6.6–11.5)
Doctor has not recommended it	5.1 (2.8–9.4)	5.9 (4.1–8.4)
Plan to wait and see if it is safe and may get it later	62.2 (54.0–69.8)	56.2 (51.3–60.9)
Concerned about the cost of the vaccine	‡	3.7 (2.5–5.4)
Don’t trust COVID-19 vaccines	23.7 (17.5–31.2)	21.9 (18.9–25.2)
Don’t trust the government	19.0 (11.1–30.7)	18.4 (15.1–22.2)
Don’t think COVID-19 is that big of a threat	‡	5.2 (3.6–7.6)
It's hard for me to get a COVID-19 vaccine	‡	4.4 (2.7–7.1)
Other	12.5 (9.3–16.5)	11.8 (9.2–15.1)
Probably not/definitely not		
Concerned about possible side effects	51.2 (45.4–56.9)	54.9 (51.5–58.2)
Don’t know if a vaccine will protect me	21.3 (17.0–26.4)	24.4 (21.7–27.2)
Don’t believe I need a vaccine	36.6 (30.8–42.9)	31.0 (28.8–33.3)
Doctor has not recommended it	15.3 (11.2–20.4)†	9.4 (7.9–11.2)
Plan to wait and see if it is safe and may get it later	26.8 (22.6–31.5)	28.3 (25.7–31.0)
Concerned about the cost of the vaccine	‡	3.6 (2.3–5.4)
Don’t trust COVID-19 vaccines	50.8 (45.1–56.5)	50.9 (48.0–53.7)
Don’t trust the government	39.4 (33.8–45.3)	43.1 (40.4–45.9)
Don’t think COVID-19 is that big of a threat	21.9 (17.4–27.2)	24.6 (22.6–26.7)
It's hard for me to get a COVID-19 vaccine	‡	2.5 (1.4–4.2)
Other	30.1 (25.0–35.8)†	21.7 (19.2–24.4)
Only received 1 of 2 doses		
I believe one dose is enough to protect me	‡	‡
I experienced side effects from the dose of COVID-19 vaccine I received	51.2 (32.6–69.4)	34.2 (24.0–46.0)

*All percentages are weighted. COVID-19, coronavirus disease. †Significant at p<0.05 comparing ever and never had previous COVID-19 diagnosis. ‡Estimates suppressed if relative standard error >30%.

## Discussion

Despite the availability of COVID-19 vaccines in the United States, vaccination coverage has plateaued since May 2021, and uptake remains suboptimal (*19*). Although the exact proportion of the population that must be vaccinated to attain herd immunity is debated (*20*,*21*), the current full vaccination coverage estimates found in this study (69% and 82% among those who ever or never had COVID-19, lower in some subpopulations) is probably insufficient to prevent ongoing community transmission (*20*,*21*). We found that those who have had COVID-19 were less likely to have been vaccinated with >1 dose or to be fully vaccinated and were more likely to be reluctant to get a future vaccination than were those who had not had the illness. This finding suggests a lack of understanding about the duration of immunity conferred by infection, as well as concerns about vaccine-associated side effects, general vaccine safety, and a lack of trust in the government. Addressing concerns about possible side effects as well as encouraging providers to have discussions about the safety and importance of vaccination may be critical for increasing coverage among this group. Regardless of sociodemographic characteristics, those who had a previous diagnosis of COVID-19 were also more likely to report uncertainty (probably/unsure) or reluctance (probably will not/definitely will not) toward vaccination. These results highlight the value of increasing vaccine uptake and confidence among those who have had the infection, particularly as new variants of SARS-CoV-2 emerge.

We found that prevalence of COVID-19 diagnoses and vaccination levels vary widely across states. In states with lowest vaccination coverage, prevalence of cases was highest. Previous studies have shown that persons in the South and Midwest were less likely to be vaccinated than were those in other areas of the United States (*9*) and that disparities in COVID-19 vaccination and intentions to be vaccinated persist among racial/ethnic groups, adults with lower incomes, and those living in rural areas (*22**–**24*). It is likely that states with low vaccination coverage have a higher proportion of persons who are hesitant toward vaccination, have higher social vulnerability, or have more access barriers (*22**–**24*). These findings point to the value of reaching these pockets of vulnerability, where the likelihood of future outbreaks is high (*25*), especially because new variants of SARS-CoV-2, such as the Delta variant, spread more easily and quickly than other variants, which may lead to increased cases, hospitalizations, and deaths from COVID-19 (*26*). CDC stated that the 3 authorized vaccines (Pfizer-BioNTech, Moderna, and J&J/Janssen) effectively protect against the circulating variants (*10*,*27*). The public should be informed about the need for vaccination despite a history of COVID-19 infection because it remains uncertain whether infection confers immunity and, if so, the duration of protection.

Adding to the literature, we found that reluctance to get vaccinated (i.e., those who probably will not or definitely will not get vaccinated) was higher among all adults who ever had COVID-19 and by most socioeconomic characteristics. Reluctance to get vaccinated was highest among adults who were younger, identified as part of a non-Hispanic other racial/ethnic group, had lower educational attainment or lower household income, had no health insurance, lived in larger households, and lived in other transient settings (e.g., mobile homes, boats, vans, or recreational vehicles). These results suggest the need to focus interventions on groups already vulnerable to infection from COVID-19, including those living in larger households (which may be multigenerational) and adults living in transient homes.

The first limitation of our study involves representativeness of the sample. Although sampling methods and data weighting were designed to produce nationally representative results, respondents might not be fully representative of the general US adult population (*28*). Second, vaccination status and COVID-19 diagnosis were self-reported and subject to misclassification. Although prevalence of previous COVID-19 diagnosis for this sample was 15%, studies have found that >1 in 3 Americans had COVID-19 in 2020; this percentage is likely to be higher in August 2021, when the survey was conducted, suggesting that the survey responses may be underestimated (*29*). Third, because the HPS is a cross-sectional survey, temporal relationships between COVID-19 disease and vaccination cannot be assessed. Fourth, HPS data available to the public do not have information on county-level analyses, which would be useful for assessing vaccination coverage and intention at the local level. Fifth, the HPS response rate is low (<10%). However, nonresponse bias assessment conducted by the Census Bureau found that the survey weights adjusted for most of this bias. Although some bias may remain, we do not expect it to be sufficient to change the conclusions (*28*). Last, small sample sizes among adults who ever had COVID-19 may have contributed to lack of statistically significant results for some of the socioeconomic factors associated with being uncertain or reluctant.

Despite COVID-19 vaccines being readily available to all Americans, many persons are still hesitant about getting vaccinated. Our finding that those with previous COVID-19 infection are less likely to be vaccinated or to complete all recommended doses suggests a strong need for direct messaging that infection does not confer reliable immunity. Although current health campaigns to improve COVID-19 vaccine uptake have successfully reached many Americans, additional efforts are needed to ensure that those with a COVID-19 history are well informed of the CDC COVID-19 vaccine recommendations. Studies have found that confidence in vaccines, weaker complacency, and collective responsibility were associated with higher likelihood of COVID-19 vaccination (*30*). The CDC strategy for reinforcing confidence in COVID-19 vaccines is to build trust in the safety and efficacy of vaccines, empower healthcare personnel to recommend vaccination to their patients, and engage communities around vaccine confidence by tailoring culturally appropriate messages and materials (*31*). This information could be emphasized to patients at the time of diagnosis, during clinical visits, and reinforced at time of hospital discharge among those who have been hospitalized. Messages should include the potential for reinfection, the role that nonvaccinated persons may play in continuing community transmission, and the potential for the emergence of additional variants of concern.

In summary, adults who have had COVID-19 are less likely to have been vaccinated than those who had not had the illness, suggesting the need to better educate the public about the importance of being vaccinated, regardless of previous COVID-19 diagnosis. Promising strategies to promote vaccination in localities with low vaccine uptake include door-to-door outreach, mobile vaccination units, vaccine offerings on public transportation and at public events, and incentives such as cash or other rewards (*32*,*33*). Studies have also shown that clear and consistent messages about the safety and effectiveness of vaccines; the protection they provide for families and communities; and the value of vaccines for returning to school, work, and social activities are needed to increase uptake and boost confidence (*5*,*9*). Building confidence in COVID-19 vaccines is critical for ensuring that communities are fully vaccinated and protected from the harmful effects of COVID-19. Reinforcing the message that the COVID-19 vaccine is needed, despite previous infection, will help protect communities against further spread of the disease, particularly as new variants emerge.

Appendix Supplemental results from study of COVID-19 vaccination coverage, behaviors, and intentions among previously adults with previous diagnosis, United States.
