# Supplementary material for: COVID-19 Vaccination Coverage, Behaviors, and Intentions among Adults with Previous Diagnosis, United States
Source: Emerg Infect Dis. 2022 Mar;28(3):631–8. doi: 10.3201/eid2803.211561 (PMC8888235; doi:10.3201/eid2803.211561)
Supplement: Appendix — Supplemental results from study of COVID-19 vaccination coverage, behaviors, and intentions among previously adults with previous diagnosis, United States. [file 21-1561-Techapp-s1.pdf]

Article DOI: https://doi.org/10.3201/eid2803.211561

COVID-19 Vaccination Coverage, Behaviors, and Intentions among Adults with Previous Diagnosis, United States

Appendix

Appendix Table 1. COVID-19 vaccination coverage and intention to receive all required doses, by sociodemographic characteristics and stratified by prior COVID-19 diagnosis, United States, July 21–August 2, 2021*

	Received ≥1 dose				Received all required doses	
	Ever had COVID-19		Never had COVID-19 (referent)			
Characteristic	% (95% CI)	aPR (95% CI)	% (95% CI)	aPR (95% CI)	Ever had COVID-19, % (95% CI)	Never had COVID-19 (referent), % (95% CI)
Age group, y						
18–49 (referent)	65.9 (63.1–68.6)†	1	77.9 (76.8–78.9)	1	60.8 (58.1–63.5)†	74.5 (73.3–75.7)
50–64	81.1 (77.9–83.9)†	1.22 (1.14–1.30)	89.2 (88.2–90.1)	1.12 (1.10–1.14)	77.3 (74.3–80.0)†	86.2 (85.2–87.2)
≥65	89.4 (85.6–92.3)†	1.33 (1.23–1.43)	94.0 (93.1–94.7)	1.17 (1.15–1.19)	86.2 (82.0–89.5)†	92.0 (90.9–93.1)
Sex						
F (referent)	75.6 (73.6–77.5)†	1	84.6 (83.7–85.4)	1	70.4 (68.4–72.5)†	81.4 (80.4–82.3)
M	71.9 (68.1–75.4)†	0.93 (0.88–0.99)	84.9 (83.8–85.9)	1.01 (0.99–1.02)	68.4 (64.5–72.1)†	82.5 (81.2–83.6)
Transgender or other	58.6 (40.1–75.0)	0.81 (0.56–1.19)	75.3 (68.5–81.1)	0.98 (0.89–1.07)	51.8 (33.7–69.5)	70.7 (63.3–77.1)
Race/ethnicity						
Non-Hispanic White (referent)	72.8 (70.4–75.1)†	1	85.0 (84.3–85.8)	1	69.1 (66.7–71.4)†	82.9 (82.1–83.8)
Non-Hispanic Black	69.7 (63.3–75.3)†	0.96 (0.87–1.05)	79.1 (76.6–81.3)	0.96 (0.94–0.99)	65.3 (58.9–71.1)†	73.5 (70.7–76.0)
Hispanic	74.7 (68.6–80.0)†	1.11 (1.01–1.22)	82.7 (80.0–85.1)	1.06 (1.03–1.09)	67.9 (62.5–72.9)†	78.1 (75.2–80.8)
Non-Hispanic Asian	87.6 (79.8–92.7)†	1.21 (1.10–1.32)	95.3 (92.9–96.9)	1.11 (1.08–1.14)	83.1 (75.8–88.5)†	92.8 (90.5–94.6)
Non-Hispanic other/multiple races	71.2 (59.8–80.5)†	1.08 (0.94–1.24)	82.4 (79.1–85.3)	1.02 (0.98–1.06)	70.1 (59.1–79.2)	79.5 (76.1–82.6)
Education						
High school or less (referent)	70.4 (66.6–73.8)†	1	76.9 (75.3–78.5)	1	64.9 (61.1–68.6)†	72.7 (70.9–74.4)
Some college or college graduate	73.6 (71.1–75.9)†	1.05 (0.98–1.12)	87.1 (86.3–87.8)	1.08 (1.06–1.11)	69.6 (66.9–72.1)†	84.7 (83.9–85.5)
Above college graduate	85.0 (81.6–87.8)†	1.16 (1.08–1.25)	95.9 (95.4–96.4)	1.14 (1.11–1.17)	82.2 (78.7–85.2)†	94.7 (94.1–95.2)
Annual household income						
<$35,000 (reference)	69.3 (63.3–74.6)†	1	75.8 (74.0–77.6)	1	61.1 (55.7–66.3)†	71.1 (69.1–73.1)
$35,000–$49,999	73.7 (66.4–79.8)†	1.08 (0.96–1.22)	86.0 (83.7–88.0)	1.09 (1.06–1.13)	70.3 (63.4–76.4)†	83.1 (80.6–85.3)
$50,000–$74,999	71.6 (64.7–77.6)†	1.04 (0.92–1.17)	86.1 (84.6–87.5)	1.08 (1.05–1.11)	68.7 (61.8–74.8)†	83.4 (81.8–84.9)
≥$75,000	79.8 (77.3–82.1)†	1.14 (1.05–1.25)	91.8 (90.9–92.6)	1.13 (1.10–1.16)	77.0 (74.1–79.6)†	90.3 (89.3–91.1)
Did not report	70.6 (66.3–74.6)†	0.94 (0.78–1.15)	80.7 (79.0–82.2)	1.11 (1.07–1.15)	65.8 (61.4–69.9)†	77.3 (75.6–78.8)

Characteristic	Received ≥1 dose: Ever had COVID-19, % (95% CI)	Received ≥1 dose: Ever had COVID-19, aPR (95% CI)	Received ≥1 dose: Never had COVID-19 (referent), % (95% CI)	Received ≥1 dose: Never had COVID-19 (referent), aPR (95% CI)	Received all required doses: Ever had COVID-19, % (95% CI)	Received all required doses: Never had COVID-19 (referent), % (95% CI)
Insurance status						
Insured (reference)	75.6 (73.2–77.7)†	1	87.6 (86.9–88.2)	1	71.2 (69.0–73.4)†	85.1 (84.4–85.8)
Not insured	56.5 (44.4–67.9)	0.87 (0.70–1.08)	64.9 (61.0–68.6)	0.85 (0.80–0.91)	52.4 (41.5–63.0)	59.7 (55.6–63.7)
No. persons in household						
1–2 (referent)	81.5 (79.4–83.4)†	1	89.6 (88.9–90.2)	1	77.1 (74.7–79.4)†	87.6 (86.9–88.3)
3–5	70.4 (67.2–73.4)†	0.93 (0.88–0.99)	82.0 (80.8–83.2)	0.96 (0.94–0.97)	66.2 (63.0–69.2)†	78.6 (77.2–79.9)
≥6	65.0 (58.2–71.3)†	0.87 (0.77–0.99)	76.3 (72.8–79.4)	0.92 (0.88–0.96)	59.0 (52.1–65.6)†	71.9 (68.4–75.2)
Housing structure						
Single-family home (referent)	74.4 (71.5, 77.2)†	1	87.4 (86.5, 88.2)	1	70.2 (67.4, 72.9)†	85.1 (84.1, 86.1)
Townhouse/condo	77.0 (64.6, 86.1)	1.09 (0.96, 1.24)	86.5 (84.2, 88.5)	1.01 (0.99, 1.04)	73.7 (61.8, 83.0)	83.4 (80.6, 85.8)
Multi-unit home	71.9 (63.8, 78.8)†	1.03 (0.92, 1.15)	85.3 (83.8, 86.7)	1.03 (1.01, 1.05)	68.1 (59.6, 75.5)†	81.4 (79.7, 83.1)
Other (e.g., mobile home, boat, van, RV)	63.4 (53.1, 72.6)	0.90 (0.77, 1.06)	68.3 (63.8, 72.5)	0.86 (0.80, 0.92)	56.0 (45.9, 65.6)	63.3 (59.2, 67.3)

†All percentages are weighted. aPR = adjusted prevalence ratio; COVID-19, coronavirus disease.
†Significant at p<0.05 comparing ever and never had previous COVID-19 diagnosis by each sociodemographic characteristic.

Appendix Table 2. Factors associated with vaccination intention, stratified by prior COVID-19 diagnosis, United States, July 21–August 2, 2021*

Characteristic	Probably will get a vaccine/ unsure about getting a vaccine: Ever had COVID-19, % (95% CI)	Ever had COVID-19, aPR (95% CI)	Never had COVID-19 (referent), % (95% CI)	Never had COVID-19 (referent), aPR (95%CI)	Probably will not get a vaccine/ definitely will not get a vaccine: Ever had COVID-19, % (95% CI)	Ever had COVID-19, aPR (95% CI)	Never had COVID-19 (referent), % (95% CI)	Never had COVID-19 (referent), aPR (95% CI)
All adults, ≥18 y	10.3 (8.9–11.8)†		4.8 (4.4–5.3)		14.0 (12.5–15.6)†		9.4 (8.8–9.9)	
Age group, y								
18–49(referent)	14.0 (12.0–16.2)†	1	7.0 (6.3–7.7)	1	16.8 (14.9–19.0)†	1	13.3 (12.5–14.2)	1
50–64	6.3 (4.5–8.6)†	0.42 (0.29–0.61)	3.6 (3.0–4.3)	0.58 (0.47–0.73)	11.2 (9.0–13.8)†	0.74 (0.54–0.99)	6.5 (5.9–7.3)	0.52 (0.45–0.59)
≥65	2.4 (1.4–3.9)	0.20 (0.11–0.38)	1.6 (1.2–2.2)	0.23 (0.17–0.31)	7.6 (4.8–11.8)†	0.49 (0.31–0.76)	4.0 (3.3–4.7)	0.30 (0.23–0.38)
Sex								
F (referent)	10.4 (8.8–12.2)†	1	5.2 (4.7–5.8)	1	12.2 (11.0–13.5)†	1	8.8 (8.3–9.4)	1
M	10.6 (8.1–13.6)†	1.10 (0.79–1.52)	4.4 (3.9–5.0)	0.84 (0.71–1.00)	14.8 (12.4–17.7)†	1.25 (1.02–1.51)	9.5 (8.7–10.5)	1.04 (0.93–1.16)
Transgender or other	3.1 (1.2–7.3)	0.30 (0.09–1.01)	‡	0.27 (0.12–0.58)	32.9 (19.5–49.9)	2.74 (1.42–5.29)	19.6 (14.6–25.8)	1.81 (1.21–2.71)
Race/ethnicity								
Non-Hispanic White (referent)	8.8 (7.4–10.5)†	1	4.0 (3.6–4.5)	1	16.5 (14.7–18.6)†	1	10.2 (9.5–10.9)	1
Non-Hispanic Black	13.7 (8.9–20.5)	1.47 (0.90–2.40)	10.0 (8.1–12.2)	1.68 (1.34–2.12)	11.0 (7.6–15.5)	0.60 (0.40–0.89)	8.7 (7.4–10.2)	0.77 (0.64–0.93)
Hispanic	13.0 (9.3–17.9)†	1.27 (0.84–1.91)	5.9 (4.7–7.5)	0.89 (0.64–1.22)	10.3 (7.5–14.0)	0.49 (0.32–0.77)	8.6 (7.1–10.5)	0.54 (0.43–0.69)
Non-Hispanic Asian	‡	0.53 (0.17–1.67)	2.0 (1.2–3.4)	0.57 (0.28–1.14)	‡	0.21 (0.06–0.81)	‡	0.20 (0.08–0.51)
Non-Hispanic other/multiple races	‡	0.74 (0.28–1.94)	4.3 (3.2–5.8)	0.85 (0.61–1.19)	20.1 (11.8–32.2)	0.84 (0.48–1.46)	11.8 (9.4–14.6)	0.93 (0.69–1.27)
Education								
High school or less (referent)	12.9 (10.1–16.3)†	1	7.5 (6.5–8.6)	1	13.8 (11.2–16.9)	1	13.3 (12.1–14.6)	1

Some college or college graduate	9.4 (7.9–11.2)†	0.74 (0.51–1.07)	4.0 (3.7–4.4)	0.68 (0.54–0.84)	14.8 (13.0–16.7)†	1.15 (0.85–1.57)	8.2 (7.6–8.8)	0.68 (0.59–0.78)
Above college graduate	3.3 (2.2–5.1)†	0.32 (0.19–0.57)	0.8 (0.6–1.0)	0.17 (0.12–0.25)	11.1 (8.6–14.4)†	0.86 (0.54–1.35)	3.1 (2.6–3.7)	0.29 (0.22–0.38)
Annual household income								
<$35,000 (reference)	13.9 (9.8–19.3)†	1	8.9 (7.7–10.4)	1	15.0 (11.6–19.3)	1	12.5 (11.2–13.8)	1
$35,000–$49,999	10.7 (6.9–16.1)†	0.83 (0.48–1.43)	3.9 (3.0–5.1)	0.55 (0.40–0.74)	11.7 (8.0–17.0)	0.79 (0.52–1.19)	8.7 (7.0–10.7)	0.77 (0.62–0.96)
$50,000–$74,999	‡	0.84 (0.35–1.99)	4.4 (3.5–5.4)	0.65 (0.49–0.85)	14.0 (10.4–18.5)†	0.91 (0.63–1.33)	8.9 (7.8–10.1)	0.87 (0.75–1.01)
≥$75,000	6.0 (4.5–7.8)†	0.57 (0.35–0.94)	2.1 (1.7–2.7)	0.37 (0.28–0.49)	12.8 (10.7–15.3)†	0.75 (0.54–1.03)	5.7 (5.1–6.5)	0.61 (0.50–0.74)
Did not report	11.7 (8.7–15.4)†	1.13 (0.58–2.20)	5.7 (4.8–6.8)	0.62 (0.45–0.86)	15.2 (12.5–18.2)†	1.06 (0.68–1.65)	12.2 (10.9–13.6)	0.66 (0.51–0.87)
Insurance status								
Insured (reference)	9.5 (8.0–11.3)†	1	4.0 (3.7–4.4)	1	12.8 (11.2–14.5)†	1	7.7 (7.2–8.2)	1
Not insured	‡	1.00 (0.42–2.35)	9.0 (6.9–11.7)	1.00 (0.74–1.36)	23.3 (15.4–33.7)	1.57 (1.04–2.38)	21.0 (17.0–25.7)	1.59 (1.29–1.98)
No. persons in household								
1–2 (referent)	6.2 (5.2–7.5)†	1	3.1 (2.7–3.5)	1	10.2 (8.6–12.1)†	1	6.5 (6.0–7.1)	1
3–5	11.8 (9.4–14.5)†	1.24 (0.90–1.72)	6.0 (5.3–6.8)	1.47 (1.20–1.80)	15.7 (13.6–18.2)†	1.33 (1.00–1.76)	10.4 (9.5–11.3)	1.28 (1.11–1.46)
≥6	14.4 (9.7–20.8)†	1.32 (0.65–2.71)	6.3 (4.9–8.0)	1.44 (1.00–2.08)	16.6 (12.8–21.3)	1.41 (0.97–2.04)	16.4 (13.7–19.6)	1.78 (1.41–2.24)
Housing structure								
Single-family home (referent)	9.4 (7.7–11.5)†	1	3.6 (3.2–4.1)	1	14.4 (12.3–16.7)†	1	8.2 (7.5–9.0)	1
Townhouse/condo	‡	1.10 (0.61–1.98)	6.3 (4.7–8.3)	1.39 (1.04–1.86)	5.9 (3.4–10.0)	0.41 (0.23–0.75)	6.4 (5.0–8.0)	0.71 (0.55–0.92)
Multi-unit home	11.9 (6.5–20.7)	0.99 (0.52–1.88)	6.2 (5.1–7.6)	1.16 (0.92–1.47)	10.8 (8.4–13.9)†	0.75 (0.55–1.01)	7.0 (6.1–8.1)	0.69 (0.57–0.84)
Other (e.g., mobile home, boat, van, RV)	10.1 (6.0–16.3)	1.00 (0.59–1.67)	8.7 (6.8–11.1)	1.50 (1.11–2.04)	26.1 (17.3–37.3)	1.56 (1.03–2.37)	19.3 (16.2–22.7)	1.51 (1.23–1.84)

*All percentages are weighted, aPR, adjusted prevalence ratio; COVID-19, coronavirus disease; RV, recreational vehicle.
†Statistically significant difference
(<0.05) between respondents who ever had COVID-19 and those who never had COVID-19
‡Estimates suppressed if relative standard error >30%.
